# Spontaneous Production of Glutathione-Conjugated Forms of 1,2-Dichloropropane: Comparative Study on Metabolic Activation Processes of Dihaloalkanes Associated with Occupational Cholangiocarcinoma

**DOI:** 10.1155/2017/9736836

**Published:** 2017-05-07

**Authors:** Yu Toyoda, Tappei Takada, Hiroshi Suzuki

**Affiliations:** Department of Pharmacy, The University of Tokyo Hospital, 7-3-1 Hongo, Bunkyo-ku, Tokyo 113-8655, Japan

## Abstract

Recently, epidemiological studies revealed a positive relationship between an outbreak of occupational cholangiocarcinoma and exposure to organic solvents containing 1,2-dichloropropane (1,2-DCP). In 1,2-DCP-administered animal models, we previously found biliary excretion of potentially oncogenic metabolites consisting of glutathione- (GSH-) conjugated forms of 1,2-DCP (GS-DCPs); however, the GS-DCP production pathway remains unknown. To enhance the understanding of 1,2-DCP-related risks to human health, we examined the reactivity of GSH with 1,2-DCP in vitro and compared it to that with dichloromethane (DCM), the other putative substance responsible for occupational cholangiocarcinoma. Our results showed that 1,2-DCP was spontaneously conjugated with GSH, whereas this spontaneous reaction was hardly detected between DCM and GSH. Further analysis revealed that glutathione *S*-transferase theta 1 (GSTT1) exhibited less effect on the 1,2-DCP reaction as compared with that observed for DCM. Although GSTT1-mediated bioactivation of dihaloalkanes could be a plausible explanation for the production of reactive metabolites related to carcinogenesis based on previous studies, this catalytic pathway might not mainly contribute to 1,2-DCP-related occupational cholangiocarcinoma. Considering the higher catalytic activity of GSTT1 on DCM as compared with that on 1,2-DCP, our findings suggested differences in the activation processes associated with 1,2-DCP and DCM metabolism.

## 1. Introduction

Glutathione (GSH) is one of the most abundant antioxidative substances that regulate cellular redox homeostasis in living organisms [1, 2]. Numerous studies demonstrated the physiological importance of this tripeptide in the maintenance of human health through the elimination of endogenous reactive oxygen species, as well as the detoxification of xenobiotics. Although GSH conjugation generally represents a cell-protective process, this reaction sometimes produces cytotoxic, genotoxic, or mutagenic metabolites in the presence of several drugs, such as acetaminophen [3, 4], and industrial chemicals, such as geminal or vicinal dihaloalkanes [5, 6]. In the case of several dihaloalkanes, their observed mutagenicity might be associated with their GSH-dependent metabolism characterized by the formation of reactive metabolites, such as electrophilic sulfur mustards [5, 6].

Among the most serious human health concerns associated with exposure to dihaloalkanes is occupational cholangiocarcinoma (bile duct cancer) risk related to the chronic inhalation of 1,2-dichloropropane (1,2-DCP), an industrial chemical that requires careful handling [7–9]. An outbreak of this malignant cancer was previously reported in the printing factories, where long-term daily use of 1,2-DCP-enriched (>98%) cleaning solvent was common. Based on these incidents, the carcinogenic hazard associated with 1,2-DCP was reclassified into group 1 (carcinogenic to humans) from group 3 (not classifiable as to its carcinogenicity to humans) by the International Agency for Research on Cancer in 2014 [8]. Additionally, chronic exposure to dichloromethane (DCM), which belongs to group 2A (probably carcinogenic to humans), is recognized as the other putative occupational-cholangiocarcinoma risk factor, given the use of DCM-containing cleaning solvent in the same and/or other printing factories [10, 11]. Hence, we have focused on these two occupational cholangiocarcinoma-associated dihaloalkanes that have a similar chemical structure with two chloride atoms in a molecule.

Although the biological mechanisms of halogenated hydrocarbon-dependent carcinogenesis in bile duct are not fully understood, we have previously discovered the potentially oncogenic metabolites of 1,2-DCP in bile from rodents [12]. Untargeted metabolomics and differential analysis revealed that these metabolites are GSH-conjugated forms of 1,2-DCP (GS-DCPs) and are excreted into bile from the liver by a bile canalicular membrane transporter ABCC2, which is an ATP-dependent glutathione S-conjugate efflux pump [13, 14]. Among such GS-DCPs, there exists a putative half-mustard form (GS-Cl-DCP) that contains a chloride atom derived from the parent 1,2-DCP [12]; however, the production pathway of GS-DCPs, especially GS-Cl-DCP, remains to be elucidated.

Considering that most dihaloalkanes can be enzymatically activated by either oxidation (cytochrome P450s, CYPs) or by GSH conjugation (glutathione *S*-transferases, GSTs) [6, 15], these two pathways are likely involved in 1,2-DCP metabolism. This is supported by previous studies reporting the involvement of CYP2E1 in 1,2-DCP metabolism [16, 17], and a dramatic loss of tissue GSH caused by 1,2-DCP administration [18], although the *GST* gene involved in 1,2-DCP metabolism has not been identified. On the other hand, a part of our previous study, which was conducted in nonphysiological buffer conditions, suggested that GSH could nonenzymatically bind to 1,2-DCP, resulting in GS-Cl-DCP production [12]. This implied that spontaneous conjugation of GSH to 1,2-DCP might be a third pathway of 1,2-DCP metabolism and requires further investigation to allow for better understanding of 1,2-DCP-related risks to human health.

Here, we examined the spontaneous reactivity of 1,2-DCP with GSH under physiological pH conditions and compared the results with those of DCM. Moreover, based on a suspected association between the occupational cholangiocarcinoma risk and GST theta 1 (GSTT1) expression [19], we investigated whether these reactions were affected by GSTT1. Indeed, a pathological analysis reported the expression of this enzyme in the biliary tract of healthy human subjects, as well as those afflicted with occupationally cholangiocarcinoma [19]. Our results showed that 1,2-DCP was spontaneously conjugated with GSH and that GSTT1 exhibited less effect on this in vitro conjugation process as compared with that involving DCM, suggesting a different carcinogenic process between 1,2-DCP- and DCM-related cholangiocarcinoma.

## 2. Materials and Methods

### 2.1. Materials

The following compounds were purchased from commercial sources indicated in parentheses: 1,2-DCP (99%, Lot number 01113DOV; Sigma-Aldrich, St. Louis, MO, USA); DCM (99%, Lot number V2H8691) and glutathione-reduced form (Nacalai Tesque Inc., Kyoto, Japan); 5,5′-dithiobis(2-nitrobenzoic acid), known as Ellman's reagent (DTNB) [20], and 1-chloro-2,4-dinitrobenzene (CDNB; Wako Pure Chemical Industries Ltd., Osaka, Japan); 0.1% formic acid in water (*v*/*v*), Solvent Blends and 0.1% formic acid in acetonitrile (*v*/*v*), Solvent Blends in Optima LC/MS grade (ThermoFisher Scientific K.K., Yokohama, Japan); human GSTT1 protein [1 mg/mL, in 20 mM Tris-HCl buffer (pH 8.0) containing 10% glycerol] (ATGP0346; ATGen Co. Ltd., South Korea); and 1,2-epoxy-3-(4-nitrophenoxy) propane (EPNP; Santa Cruz Biotechnology, Santa Cruz, CA, USA). All other chemicals used were commercially available and of analytical grade.

### 2.2. In Vitro Reaction of Halogenated Hydrocarbons with GSH

An aliquot of 1,2-DCP or DCM was added into 100 mM potassium phosphate buffer (pH 7.4) containing GSH (at a final concentration of 6 mM) at the indicated final concentrations (*v*/*v*). For enzymatic assays, GSTT1 protein solution (50 *μ*g/mL final concentration) or control solution [20 mM Tris-HCl buffer (pH 8.0) containing 10% glycerol] was mixed with the potassium buffer containing GSH, followed by addition of each halogenated hydrocarbon to the reaction mixture. The mixture was then sealed, vortexed well, and incubated at 37 °C. After the indicated period, the reaction mixture was placed on ice and immediately subjected to GSH quantification assay and LC-MS analysis. The details are described in subsequent sections.

### 2.3. GSH Quantification Assay

GSH concentrations were measured spectrophotometrically with DTNB as described in our previous study [12] with minor modifications. After dilution with nine volumes of water, a 10 *μ*L aliquot of each sample was mixed with 150 *μ*L of DTNB solution [0.5 mM DTNB in 0.1 M sodium phosphate buffer (pH 6.5)] and incubated for 10 min at room temperature. The resulting absorbance at 412 nm was obtained using a Varioskan flash microplate reader (ThermoFisher Scientific K.K.), and a standard curve for quantification was generated using GSH solutions of known concentration.

### 2.4. Analytical Sample Preparation and LC-MS Analysis

Each reaction mixture was treated with four volumes of methanol, and the mixture was vortexed well for 2 min and centrifuged at 15,000 ×g for 10 min at 4 °C. The resulting supernatant was transferred to a new glass vial and subjected to ultraperformance liquid chromatography separation, followed by untargeted metabolomics analysis according to our previous report [12]. Briefly, analysis of all samples was performed by a Thermo Scientific-Q Exactive Orbitrap System (ThermoFisher Scientific K.K.) for high-resolution MS scanning coupled with a DIONEX Ultimate 3000 Rapid Separation LC system (ThermoFisher Scientific K.K.). Sample (2 *μ*L) was injected onto a Syncronis aQ column (100 × 21 mm, 5 *μ*m; ThermoFisher Scientific K.K.) and separated using the following gradient mobile phases consisting of 0.1% formic acid in water (A) and 0.1% formic acid in acetonitrile (B) at a flow rate of 300 *μ*L/min: 0 to 5 min, 0% B; 5 to 10 min, 0% to 90% B; 10 to 30 min, 90% B; and 30 to 40 min, 0% B. A heated electrospray ionization (ESI) probe in positive ion mode was used for the ionization. Full MS scans were operated in full-spectrum acquisition mode from *m*/*z* 100 to 800, with a resolution of 70,000 FWHM at *m*/*z* 200. Detection was performed using a Q Exactive mass spectrometer controlled by Excalibur software (ThermoFisher Scientific K.K.), and exact mass calculation and peak analysis were performed using the Qualbrowser program (ThermoFisher Scientific K.K.). Additionally, solvent blank was also measured to assess background peaks that were subtracted during mass spectrum analysis.

### 2.5. Determination of GSTT1 Enzyme Activity toward EPNP

EPNP, a GSTT-specific substrate [21], was used to determine GSTT1 enzyme activity. According to previous reports [22, 23], we examined the production of the EPNP-glutathione conjugate photometrically in the presence or absence of GSTT1. Briefly, 100 *μ*L of reaction mixture consisting of 100 mM potassium phosphate buffer (pH 6.5, prewarmed to 37 °C), 6 mM GSH, and 0.5 mM EPNP (31.25 mM stock in ethanol) was prepared. Immediately after the addition of 5 *μ*L of GSTT1 solution, absorption at 360 nm in the well-mixed reaction mixture was measured using an ultraviolet spectrophotometer (UV-1800; Shimadzu Corp., Kyoto, Japan.) in time-scanning mode. A complete assay mixture without GSTT1 protein was used as a reference control. Enzyme activity was calculated based on the previously determined molar extinction coefficient (Δ*ε*_360_ = 0.5 mM^−1^ cm^−1^) [22].

### 2.6. Differential Analysis

To detect differences in chemical substances between GSTT1-treated and GSTT1-untreated samples, we performed differential analysis of profiling data using SIEVE 2.1 software (ThermoFisher Scientific K.K.). Briefly, to subtract background peaks and perform peak integration and grouping as components, acquisition data from the samples and solvent blanks were processed by SIEVE 2.1 using Components Extraction, a signal-detection algorithm for nontarget analysis. Peak detection and retention-time correction were performed using the following parameters: mass range of 100 to 800 *m*/*z*, mass tolerance of 10 ppm, retention-time range of 0.01 to 30 min, and threshold for intensity of 30,000. To find the GSTT1-mediated metabolites of 1,2-DCP or DCM, we focused on peaks exhibiting intensities that increased in the GSTT1-treated group. Analytical workflow is described in detail in the Results and Discussion. The stability and reproducibility of the analytical results of differential analysis were confirmed by both individual peak analysis using the Qualbrowser program and another dataset derived from samples prepared on a different day as independent experiments. To determine the relative levels of selected metabolites based on their peak height, we generated extracted ion chromatograms based on accurate masses using a mass-extraction window of 2 ppm, followed by peak integration using the Qualbrowser program.

### 2.7. Statistical Analysis

All statistical analyses were performed by using Microsoft Excel 2013 (Microsoft Corp., Redmond, WA, USA) with Statcel3 add-in software (OMS Publishing Inc., Saitama, Japan) as described in a previous study [24]. Different statistical tests were used for different experiments and are described in the figure legends. The significance of each value was determined when *P* value was less than 0.05 and 0.01.

## 3. Results and Discussion

### 3.1. 1,2-DCP Spontaneously Reacts with GSH under Physiological pH Conditions

**Figure 1:**
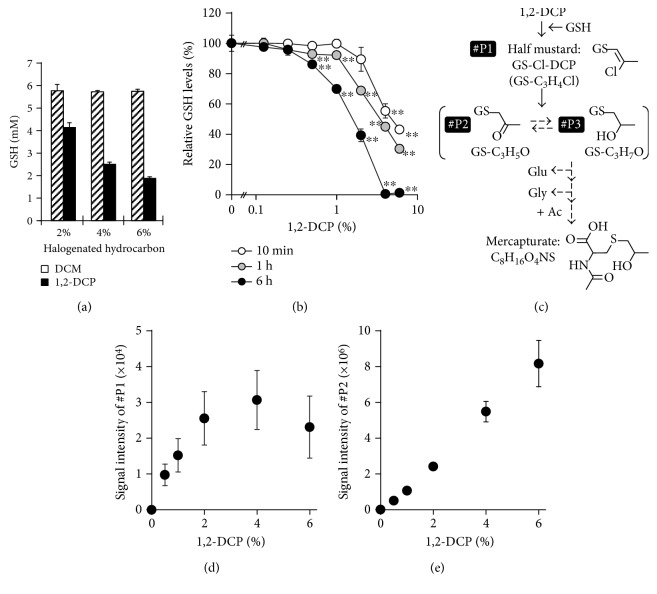
Spontaneous reaction of 1,2-DCP with GSH under physiological pH conditions. (a) GSH levels in the reaction mixture containing halogenated hydrocarbons. Each in vitro mixture containing 6 mM GSH and DCM (shaded) or 1,2-DCP (black) at the indicated concentration (*v*/*v*) was incubated at 37 °C for 60 min, followed by determination of GSH concentration in the resulting solution. (b) Time- and dose-dependent decreases in GSH concentration in the presence of 1,2-DCP. The levels of remaining GSH under each experimental condition were normalized against the initial GSH concentration. Statistical analyses for significant differences were performed according to Bartlett's test, followed by Shirley-Williams' multiple-comparison test. ^∗∗^ *P* < 0.01 versus control in each group. (c) Putative metabolic pathway of 1,2-DCP and structures of relating metabolites in the presence of GSH. This metabolic scheme was modified from that of a previous report [12]. #P1 (GS-Cl-DCP) and #P2 represent the detected GSH-conjugated forms of 1,2-DCP, whereas #P3 was not detected in the present study. Dashed arrows mean that the processes were not observed in the present study. (d and e) Dose-dependent increases in GSH-conjugated forms of 1,2-DCP. Each mixture containing 6 mM GSH and 1,2-DCP at the indicated concentration (*v*/*v*) was incubated at 37 °C for 60 min, followed by examination of (d) GS-Cl-DCP (#P1) and (e) GS-DCP (#P2) levels in the resulting solution analyzed by LC-MS. Data were expressed as means ± SD, *n* = 4.

To examine whether 1,2-DCP reacts with GSH under physiological conditions, we addressed metabolic changes in the reaction mixture (pH 7.4, 37 °C) containing 1,2-DCP (≦6%) and GSH (6 mM). First, we measured the GSH levels in the resulting solutions following a 1 h incubation at 37 °C (Figure 1(a)). Our results showed that GSH concentration was decreased in a 1,2-DCP-dependent manner, suggesting the spontaneous reaction of 1,2-DCP with GSH; however, this decrease in GSH concentration was not observed in the DCM reaction (Figure 1(a)). We subsequently confirmed that GSH concentration decreased in the presence of 1,2-DCP in time- and dose-dependent manners (Figure 1(b)). We confirmed that this decrease of GSH could not be due to the production of glutathione disulfide, an experimentally unavoidable oxidation form of GSH.

Because our previous study suggests the spontaneous production of GS-Cl-DCP in vitro [12], we focused on the 1,2-DCP metabolic pathway, including formation of the putative-carcinogenic metabolite from 1,2-DCP to GS-Cl-DCP, and then to other GS-DCPs until finally reaching its mercapturate form (Figure 1(c)). Two GSH-conjugated forms of 1,2-DCP (#P1, GS-Cl-DCP; #P2, GS-DCP) were detected in the reaction mixture following a 1 h incubation at 37 °C (Figures 1(d) and 1(e)). Meanwhile, other biliary-excreted GS-DCPs, such as #P3, or further metabolites in the mercapturate formation were not detected. Considering that there was no enzyme in the reaction mixture, most of #P3 might be produced in catalytic manner in vivo as well as the mercapturate formation. Although the analysis of each metabolite was limited to relative quantification due to the lack of corresponding standard materials, the obtained data suggested that nonenzymatic GSH conjugation contributed to the production of GS-Cl-DCP.

Additionally, the decrease in GSH concentration (Figure 1(b)) and the production of #P1 and #P2 (Figures 1(d) and 1(e)) were detectable in the incubation mixture containing at least 1% 1,2-DCP following a 1 h incubation at 37 °C. On the other hand, at high dose condition of 1,2-DCP (≧4%), the linear fashion of 1,2-DCP-dependent increase of #P1 was not observed contrary to the case of #P2. Although the mechanism explaining this difference between #P1 and #P2 was not elucidated in the present study, these results suggested that such excess amount of 1,2-DCP may affect the experimental system. Therefore, further experiments were performed using reaction mixtures containing 1% 1,2-DCP subjected to 1 h incubation at 37 °C.

### 3.2. The Effect of GSTT1 on Metabolic Profiling in the Incubation Mixture Containing GSH and 1,2-DCP or DCM

We then focused on the effect of GSTT1 on metabolic profiling in the reaction mixture containing 1,2-DCP and DCM in vitro, given the reported association between halogenated hydrocarbon-related occupational cholangiocarcinoma risk and GSTT1 expression [19]. Additionally, DCM bioactivation is reportedly catalyzed by GSTT1, resulting in the generation of the putative genotoxic intermediate, *S*-chloromethylglutathione [25, 26]. Prior to metabolic experimentation involving halogenated hydrocarbons, we confirmed the GSTT1 enzymatic activity in vitro. In the presence of GSTT1 protein, GST activity toward EPNP, a well-known GSTT1 substrate [21], was detected (125 ± 23 nmol/mg protein/min). However, GST activity toward CDNB, which is a substrate of GSTs other than GSTTs [21], was not detected, indicating minimal contamination of other GSTs in the GSTT1 enzyme solution that we used.

**Figure 2:**
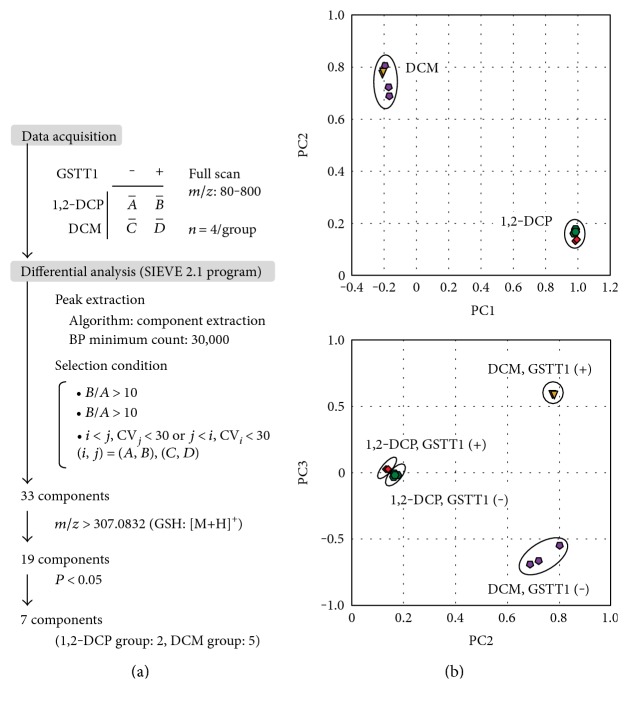
Effect of GSTT1 on the reactivity of halogenated hydrocarbon with GSH. (a) Data-processing scheme for differential analysis of four cases: in vitro reaction of 1,2-DCP or DCM with GSH in the presence or absence of GSTT1. The seven component products are summarized in Table 1. (b) Principal component analysis (PCA) based on differentially detected peaks across incubation component conditions. *Green*, 1,2-DCP without GSTT1; *red*, 1,2-DCP with GSTT1; *purple*, DCM without GSTT1; and *yellow*, DCM with GSTT1. This PCA of acquired data, such as peak information including integrated intensity, was performed by using the SIEVE 2.1 program. *Upper panel*, the first second-principal components; *lower panel*, the third two-principal components of all extracted data are plotted. *n* = 4 in each group.

To examine the GSTT1-dependent conversion of halogenated hydrocarbons, metabolic differences among samples were investigated. Untargeted data acquisition and differential analysis of four groups (A, 1,2-DCP without GSTT1; B, 1,2-DCP with GSTT1; C, DCM without GSTT1; and D, DCM with GSTT1) were conducted according to the scheme described in Figure 2(a).

To obtain a whole picture of the metabolic differences between each sample group, we first performed principal component analysis (PCA) using the SIEVE 2.1 program. Score plots of PCA based on LC-MS analysis of the incubation mixture in the presence or absence of GSTT1 are shown in Figure 2(b). As shown in the upper panel in Figure 2(b), groups containing 1,2-DCP and DCM were clearly separated on the PCA score plot (PC1 versus PC2), whereas groups in the presence and absence of GSTT1 were not well separated. On the other hand, the lower panel in Figure 2(b) shows that the score plot associated with PC3 clearly separated the subgroups containing DCM, but not 1,2-DCP group. These results suggested that GSTT1-dependent metabolic changes in the DCM group were larger than those observed in the 1,2-DCP group. Considering that the major metabolites in the simple incubation system containing GSTT1 should be GSH-conjugated halogenated hydrocarbons, the catalytic effect of GSTT1 on the GSH conjugation to 1,2-DCP might be smaller than that to DCM.

To select components (peaks) corresponding to GSTT1-dependent metabolites, we further processed the data using differential analysis via the SIEVE 2.1 program according to the following criteria (Figure 2(a)) for the initial selection of all extracted peaks: (1) peaks exhibiting obvious increases in the presence of GSTT1 based on a peak-intensity ratio (GSTT1-present group/GSTT1-absent group) > 10 and (2) peak changes in the presence of GSTT1 and a coefficient of variation (standard deviation/mean) < 30. Because the molecular mass of GSH-conjugated substances should be higher than that of GSH, we selected the peaks with *m*/*z* values higher than those of GSH during ESI-positive ionization. Our analyses revealed seven components representing significantly increased metabolites in the presence of GSTT1 (Table 1).

### 3.3. GSTT1 Enhances GSH Conjugation to DCM to a Greater Degree than to 1,2-DCP

Because differential analysis revealed that two and five components increased in the presence of GSTT1 in the 1,2-DCP and DCM groups, respectively (Table 1), we investigated all seven components by analyzing each mass chromatogram using the Qualbrowser program to screen for differential peaks.

**Figure 3:**
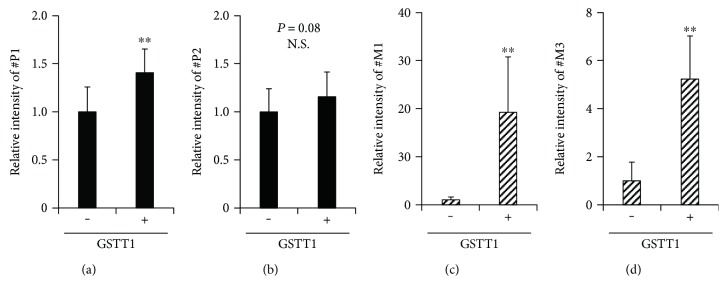
Relative levels of halogenated hydrocarbon metabolites in the presence of GSTT1. (a) #P1, (b) #P2, (c) #M1, and (d) #M3. Reaction mixtures containing 1% (*v*/*v*) (a and b) 1,2-DCP or (c and d) DCM in the presence or absence of GSTT1 protein (50 *μ*g/mL) were incubated at 37 °C for 60 min, followed by semiquantification of the levels of each metabolite in the resulting solution. Data were normalized against those acquired in the absence of GSTT1 conditions. Data were expressed as means ± SD, *n* = 12. Statistical analyses for significant differences were performed using Student's *t*-test. ^∗∗^*P* < 0.01. N.S.: not significantly different among groups.

For the 1,2-DCP group, the two components discovered during the differential analysis corresponded to GS-Cl-DCP (#P1) and GS-DCP (#P2), respectively (Table 1). Semiquantitative analyses indicated that #P1 levels in the presence of GSTT1 were slightly higher than those of the control (Figure 3(a)) and that #P2 levels did not differ significantly from those of the control (Figure 3(b)). These results suggested that a spontaneous reaction could have a much greater impact on the production of GS-Cl-DCP as compared with a GSTT1-mediated reaction.

For the DCM group, five components (#M1–5) were discovered (Table 1), and #M2 constituted the ^13^C isotope peak of #M1. As described below, some of them might be novel DCM metabolites, since these components are not likely to be derived from known metabolites in the GST-mediated metabolic pathway of DCM. Elemental analyses based on the obtained accurate mass suggested that the composition formulae for #M1 and #M3 were C_11_H_17_O_6_N_3_S and C_11_H_19_O_6_N_3_S, respectively (Table 1), which are similar to that of GSH (C_10_H_17_O_6_N_3_S). Considering that the simple incubation of DCM and GSH resulted in generation of #M1 and #M3, these two components were expected to be GSH-conjugated forms of DCM; however, our results indicated that only #M3 might constitute an *S*-methyl glutathione. Moreover, #M1 and #M3 levels in the presence of GSTT1 were higher than those observed in the control (Figures 3(c) and 3(d)). Additionally, similar analysis revealed that #M4 and #M5 were also more abundant in the presence of GSTT1 than their levels observed in the control (data not shown), although it was difficult to interpret whether #M4 and #M5 were GSH-conjugated forms based on the low degree of similarity between the formula for GSH and those of the putative GSH-conjugates (Table 1).

Although we were unable to identify the chemical structures of #M1 and #M3, they might constitute novel DCM metabolites, given that their formulae have not been reported to the best of our knowledge in the GST-mediated metabolic pathway of DCM [25, 26]. In the reported metabolic pathway, DCM is finally converted to a formic acid through intermediates, such as *S*-chloromethylglutathione (GS-CH_2_Cl) and *S*-hydroxymethylglutathione (GS-CH_2_OH). With regard to these reported intermediates, we investigated the corresponding peaks in all obtained data based on the accurate mass information. The results showed that *S*-chloromethylglutathione (*m*/*z*: 356.0677 as [M + H]^+^) was not detected. On the other hand, putative *S*-hydroxymethylglutathione (*m*/*z*: 338.1016 as [M + H]^+^) was detected in GSTT1-containing samples as a weak peak (retention time: 1.03 min) with an intensity lower than the threshold for differential analysis. These results suggested that the GSTT1 was active in incubation mixtures containing halogenated hydrocarbons.

### 3.4. Physiological Relevance of Spontaneous Reaction between 1,2-DCP and GSH and Future Perspectives

In the present study, 1,2-DCP reacted with GSH nonenzymatically under the physiological buffer conditions, suggesting the possibility of spontaneous reaction between 1,2-DCP and GSH in the liver of subjects with chronic exposure to 1,2-DCP. In addition to the involvement of CYP2E1 in 1,2-DCP metabolism [16, 17], the suspected bioactivation of 1,2-DCP mediated by GSTT1 was previously hypothesized as a plausible explanation of the production of reactive metabolites related to the occupational cholangiocarcinoma [19]. Although the physiological contributions of these two enzymes to 1,2-DCP metabolism in humans have not been elucidated, the findings in this study suggested the importance of the spontaneous reaction involving GSH as another pathway for consideration in the metabolic conversion related to 1,2-DCP. Additionally, this could plausibly explain the substantial decrease (>80%) in GSH in the liver and blood that is simultaneously observed immediately following 1,2-DCP administration to rats [18]. Considering a recent report showing that the distribution of major GSTs could not explain the species differences in susceptibility to 1,2-DCP [27], the spontaneous reaction between 1,2-DCP and GSH may contribute to the occupational cholangiocarcinoma risk. In vivo studies using animal models genetically deficient for the specific GST and/or CYP activities would be useful for further investigations. In addition, a previous study focusing on rat urinary metabolites of 1,2-DCP did not have a positive position regarding episulfonium ion formation [28]. Considering that the occupational cancer never arose in the kidney, together with the biliary excretion of the hepatic 1,2-DCP metabolite containing chloride atom [12], there should be some differences about 1,2-DCP metabolism and/or further processing between the kidney and the hepatobiliary system.

Our result showed for the first time the presence of spontaneous reaction between 1,2-DCP and GSH under physiological buffer conditions. This finding associated with dihaloalkane metabolism might also provide insight into the importance of spontaneous GSH conjugation in the metabolism of certain xenobiotics. Processes involving nonenzymatic GSH conjugation have received far less attention relative to GST-mediated processes, based on the conduct of many studies based on the physiological importance of GST proteins as metabolic enzymes in living organisms. As a result, the literatures describing GST-mediated GSH conjugations exceeds the available information regarding spontaneous GSH conjugation of xenobiotics. However, our results are supported by previous studies reporting the nonenzymatic formation of a conjugate (GS-menadione) from menadione (vitamin K_3_) and GSH in vitro, which was accompanied by the formation of aggressive oxygen species [29]. Additionally, in vitro spontaneous reaction of benzoquinones [30, 31] and 15-deoxy-Δ^12,14^-prostaglandin J_2_ [32, 33] with GSH has been documented, and recently, the involvement of nonenzymatic GSH conjugation in conversion of microcystins (toxins produced by cyanobacteria) was reported [34]. Furthermore, a report from the pharmaceutical industry showed that TAK-242, which was an antisepsis drug candidate, spontaneously reacted with GSH [35]. Because certain chemicals exhibit high reactivity with GSH, nonenzymatic GSH conjugation deserves additional study to elucidate a complete picture of xenobiotic metabolism.

## 4. Conclusions

In summary, our findings revealed that 1,2-DCP was capable of spontaneously reacting with GSH under physiological pH conditions and that GSTT1 exhibited less effect on this process as compared with its effect on DCM, which exhibited minimal spontaneous reactivity with GSH. Although 1,2-DCP and DCM are both dihaloalkanes, the latent molecular basis for cholangiocarcinogenesis may differ between these two chemicals. Based on these results, additional research is needed to acquire a deeper understanding of halogenated hydrocarbon-related carcinogenesis.

## Figures and Tables

**Table 1:** List of seven components picked up by differential analysis.

Halogenated hydrocarbon	ID	*m*/*z* (positive)	Retention time (min)	Formula	Theoretical *m*/*z* [M + H]^+^	GSTT1 (+) versus GSTT1(−)	
						Ratio	*P* value
1,2-DCP	#P2	364.1172	3.2	C13H21O7N3S	364.1173	1.15	0.036
	#P1	382.0835	8.3	C13H20O6N3SCl	382.0834	2.18	0.000
DCM	#M1	320.0911	2.4	C11H17O6N3S	320.0911	>10	0.032
	#M2	321.0944		Isotope peak of #M1		>10	0.031
	#M3	322.1068	3.0	C11H19O6N3S	322.1067	>10	0.005
	#M4	418.0680	0.9	C15H24ON3P5	418.0680	>10	0.008
	#M5	493.9794	0.9	C18H5O7N7P2	493.9798	>10	0.002

1,2-DCP: 1,2-dichloropropane; DCM: dichloromethane; GSTT1: glutathione *S*-transferase theta 1. Component IDs #P2 and #P1 correspond to the same ones in Figure 1(c), respectively. Ideal *m*/*z* as [M + H]^+^ in positive ion mode was calculated based on the monoisotopic mass of corresponding formula. Ratio and *P* value were determined by using the SIEVE 2.1 program.
